# 9-Ethynyl-1,2-dimethyl-1,2-dicarba-*closo*-dodeca­borane (1,2-Me_2_-9-HC C-*closo*-1,2-C_2_B_10_H_9_)

**DOI:** 10.1107/S1600536810022440

**Published:** 2010-06-18

**Authors:** Maik Finze, Guido J. Reiss

**Affiliations:** a Institut für Anorganische Chemie und Strukturchemie II, Heinrich Heine Universität Düsseldorf, Universitätsstrasse 1, D-40225 Düsseldorf, Germany

## Abstract

The asymmetric unit of the title compound, C_6_H_16_B_10_, contains one mol­ecule that is close to possessing a non-crystallographic plane of mirror symmetry in the space group *Pna*2_1_. The orientation of the mol­ecules in the ortho­rhom­bic cell shows that the structure can not be described in the space group *Pnma*, which has the same systematic absence conditions. The long inner-cluster C—C distance of 1.510 (5) Å is typical for {1,2-Me_2_-*closo*-1,2-C_2_B_10_} derivatives.

## Related literature

For a general overview of the functionalization of dicarba-*closo*-dodeca­boranes, see: Bregadze (1992 ▶); Kalinin & Ol’shevskaya (2008 ▶). For the synthesis and properties of {*closo*-1,2-C_2_B_10_} clusters with ethynyl groups bonded to boron, see: Zakharkin *et al.* (1981 ▶); Himmelspach & Finze (2010*a* ▶). For structures of related icosa­hedral boron cages with alkynyl groups bonded to boron, see: Finze (2008 ▶); Himmelspach & Finze (2010*b* ▶). For intensity statistics of Friedel opposites for all non-centrosymmetric space groups, see: Shmueli *et al.* (2008 ▶). 
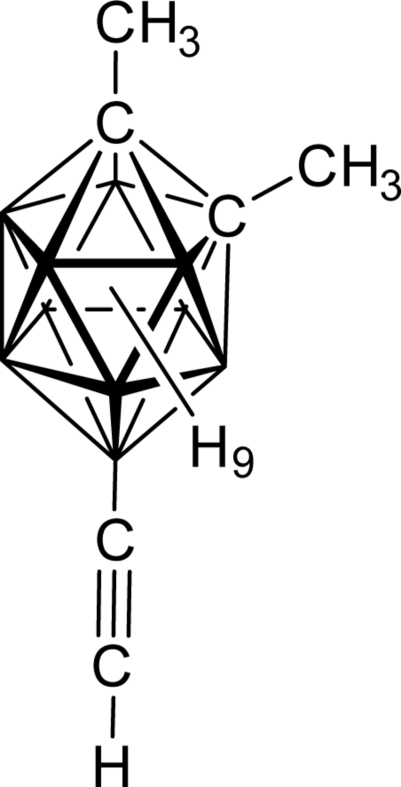


## Experimental

### 

#### Crystal data

C_6_H_16_B_10_
*M* *_r_* = 196.29
Orthorhombic, 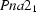

*a* = 14.5368 (8) Å
*b* = 7.0085 (3) Å
*c* = 12.5373 (5) Å
*V* = 1277.32 (10) Å^3^
*Z* = 4
Mo *K*α radiation
μ = 0.05 mm^−1^
*T* = 290 K
0.4 × 0.2 × 0.2 mm

#### Data collection

Oxford Diffraction Xcalibur Eos diffractometer
11327 measured reflections
1181 independent reflections
1049 reflections with *I* > 2σ(*I*)
*R*_int_ = 0.042

#### Refinement

*R*[*F* ^2^ > 2σ(*F* ^2^)] = 0.056
*wR*(*F* ^2^) = 0.104
*S* = 1.06
1181 reflections
150 parameters
1 restraint
H-atom parameters constrained
Δρ_max_ = 0.14 e Å^−3^
Δρ_min_ = −0.18 e Å^−3^

### 

Data collection: *CrysAlis PRO* (Oxford Diffraction, 2009 ▶); cell refinement: *CrysAlis PRO*; data reduction: *CrysAlis PRO*; program(s) used to solve structure: *SHELXS97* (Sheldrick, 2008 ▶); program(s) used to refine structure: *SHELXL97* (Sheldrick, 2008 ▶); molecular graphics: *DIAMOND* Brandenburg, 2010 ▶); software used to prepare material for publication: *SHELXL97*.

## Supplementary Material

Crystal structure: contains datablocks I, global. DOI: 10.1107/S1600536810022440/si2268sup1.cif

Structure factors: contains datablocks I. DOI: 10.1107/S1600536810022440/si2268Isup2.hkl

Additional supplementary materials:  crystallographic information; 3D view; checkCIF report

## Figures and Tables

**Table:** 

C1—C3	1.510 (5)
C1—C2	1.680 (5)
C1—B4	1.684 (6)
C1—B5	1.693 (6)
C1—B3	1.714 (6)
C1—B6	1.728 (6)
C2—C4	1.529 (5)
C2—B11	1.670 (6)
C2—B7	1.697 (6)
C2—B6	1.709 (6)
C2—B3	1.717 (5)
B3—B7	1.758 (6)
B3—B8	1.758 (6)
B3—B4	1.760 (6)
B4—B5	1.755 (6)
B4—B8	1.767 (6)
B4—B9	1.769 (6)
B5—B9	1.759 (6)
B5—B10	1.766 (6)
B5—B6	1.778 (6)
B6—B11	1.750 (7)
B6—B10	1.751 (6)
B7—B11	1.760 (6)
B7—B12	1.757 (6)
B7—B8	1.776 (6)
B8—B9	1.777 (6)
B8—B12	1.781 (6)
B9—C5	1.544 (5)
B9—B12	1.780 (6)
B9—B10	1.789 (6)
B10—B11	1.765 (7)
B10—B12	1.774 (7)
B11—B12	1.752 (6)
C5—C6	1.175 (6)

**Table:** 

C6—C5—B9	178.2 (5)

**Fig. 1.**
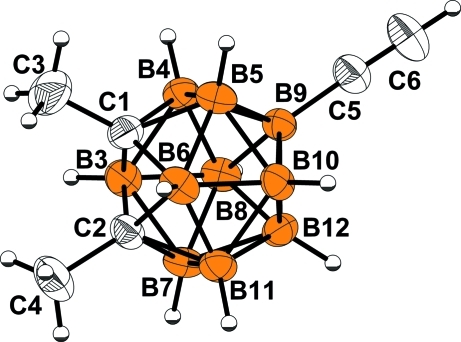
: Hydrogen atoms are drawn with arbitrary radii and the displacement ellipsoids are shown at the 35% probability level.

**Fig. 2.**
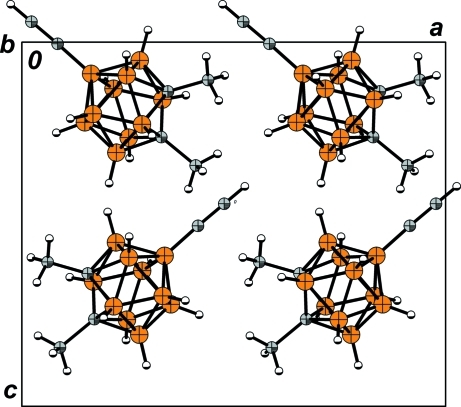
: Packing of the title compound along [0–10] showing that the non-crystallographic mirror symmetry of the molecule is not consistent with the metric and the symmetry of the true space group Pna21.

## supplementary crystallographic information

### Comment

The interest in functionalized dicarba-*closo*-dodecaboranes as building blocks for a broad range of applications is steadily increasing because of their high chemical and thermal stability as well as the diversity of their substitution patterns at the boron cage (Bregadze, 1992; Kalinin & Ol'shevskaya, 2008). The synthesis of {*closo*-C_2_B_10_} clusters with alkynyl groups bonded to boron is achieved by Pd-catalyzed Kumada-type cross-coupling reactions using the iodinated clusters and alkynyl Grignard reagents as precursors (Zakharkin *et al.*, 1981; Himmelspach & Finze, 2010*a*). The title compound 1,2-dimethyl-9-ethynyl-1,2-dicarba-*closo*-dodecaborane, which is the first structurally characterized monoethynyldicarba-*closo*-dodecaborane with a B_cluster_—C≡C—H unit, crystallizes in the orthorhombic acentric space group *Pna*2_1_ with one complete molecule in the asymmetric unit. The bond lengths and angles of the {*closo*-1,2-C_2_B_10_} cage of 1,2-Me_2_-9-HC≡C-*closo*-1,2- C_2_B_10_H_9_ are similar to those reported for the related bis(trimethylsilylalkynyl) substituted derivative 1,2-Me_2_ -9,12-(Me_3_SiC≡C)_2_-*closo*-1,2-C_2_B_10_H_9_ (Himmelspach & Finze, 2010*a*). The B—C and C≡C distances are similar to values reported for the diethynyldicarba-*closo*-dodecaboranes 9,12-(HC≡C)_2_-*closo*-1,2-C_2_B_10_H_10_ and 9,10-(HC≡C)_2_-*closo*-1,7-C_2_B_10_H_10_ (Himmelspach & Finze, 2010*a*) and the related anionic monocarba-*closo*-dodecaborate anions [12-HC≡ C-*closo*-1-CB_11_H_11_]^-^ and [7,12-(HC≡C)_2_–*closo*-1-CB_11_H_10_]^-^ (Himmelspach & Finze, 2010*b*).

### Experimental

1,2-Me_2_-9-HC≡C-*closo*-1,2-C_2_B_10_H_9_ was synthesized according to a published procedure and the spectroscopic data have been reported earlier (Himmelspach & Finze, 2010*a*). The compound was dissolved in acetonitrile and slow evaporation of the solvent resulted in colorless crystals.

### Refinement

All hydrogen atom positions were obtained from difference fourier maps. The hydrogen atoms of the methyl groups were included in the latest stages of the refinement with a riding model and for each methyl group a common *U*_iso_ value was refined. The hydrogen atoms bonded to the carborane cluster were included in the refinement with a riding model (AFIX 153) and their *U*_iso_ values were set to 1.2 of the equivalent isotropic displacement parameter of the corresponding parent atom. The hydrogen atom of the ethynyl group was positioned using a riding model (AFIX 163) and its *U*_iso_ was refined freely. In the absence of significant anomalous scattering effects, Friedel pairs were averaged, resulting in a low reflection to parameter ratio (Shmueli *et al.*, 2008).

### Crystal data

**Table:**

C_6_H_16_B_10_	*F*(000) = 408
*M**_r_* = 196.29	*D*_x_ = 1.021 Mg m^−^^3^
Orthorhombic, *P**n**a*2_1_	Mo *K*α radiation, λ = 0.71073 Å
Hall symbol: P 2c -2n	Cell parameters from 6575 reflections
*a* = 14.5368 (8) Å	θ = 3.2–27.2°
*b* = 7.0085 (3) Å	µ = 0.05 mm^−^^1^
*c* = 12.5373 (5) Å	*T* = 290 K
*V* = 1277.32 (10) Å^3^	Prism, colourless
*Z* = 4	0.4 × 0.2 × 0.2 mm

### Data collection

**Table:**

Oxford Diffraction Xcalibur Eos diffractometer	1049 reflections with *I* > 2σ(*I*)
Radiation source: Enhance (Mo) X-ray Source	*R*_int_ = 0.042
graphite	θ_max_ = 25.0°, θ_min_ = 3.2°
Detector resolution: 16.2711 pixels mm^-1^	*h* = −17→17
ω scans	*k* = −8→8
11327 measured reflections	*l* = −14→14
1181 independent reflections	

### Refinement

**Table:**

Refinement on *F*^2^	Primary atom site location: structure-invariant direct methods
Least-squares matrix: full	Secondary atom site location: difference Fourier map
*R*[*F*^2^ > 2σ(*F*^2^)] = 0.056	Hydrogen site location: inferred from neighbouring sites
*wR*(*F*^2^) = 0.104	H-atom parameters constrained
*S* = 1.06	*w* = 1/[σ^2^(*F*_o_^2^) + (0.005*P*)^2^ + 0.630*P*] where *P* = (*F*_o_^2^ + 2*F*_c_^2^)/3
1181 reflections	(Δ/σ)_max_ = 0.010
150 parameters	Δρ_max_ = 0.14 e Å^−^^3^
1 restraint	Δρ_min_ = −0.18 e Å^−^^3^

### Special details

**Table:**

Geometry. All e.s.d.'s (except the e.s.d. in the dihedral angle between two l.s. planes) are estimated using the full covariance matrix. The cell e.s.d.'s are taken into account individually in the estimation of e.s.d.'s in distances, angles and torsion angles; correlations between e.s.d.'s in cell parameters are only used when they are defined by crystal symmetry. An approximate (isotropic) treatment of cell e.s.d.'s is used for estimating e.s.d.'s involving l.s. planes.
Refinement. Refinement of *F*^2^ against ALL reflections. The weighted *R*-factor *wR* and goodness of fit *S* are based on *F*^2^, conventional *R*-factors *R* are based on *F*, with *F* set to zero for negative *F*^2^. The threshold expression of *F*^2^ > σ(*F*^2^) is used only for calculating *R*-factors(gt) *etc*. and is not relevant to the choice of reflections for refinement. *R*-factors based on *F*^2^ are statistically about twice as large as those based on *F*, and *R*- factors based on ALL data will be even larger.

### Fractional atomic coordinates and isotropic or equivalent isotropic displacement parameters (Å^2^)

**Table:**

	*x*	*y*	*z*	*U*_iso_*/*U*_eq_	
C1	0.1554 (3)	1.0151 (6)	0.6353 (3)	0.0654 (11)	
C3	0.0608 (3)	1.0836 (9)	0.6036 (4)	0.1134 (19)	
H3A	0.0361	1.1629	0.6592	0.175 (15)*	
H3B	0.0211	0.9757	0.5929	0.175 (15)*	
H3C	0.0649	1.1557	0.5387	0.175 (15)*	
C2	0.1709 (2)	0.9362 (5)	0.7606 (3)	0.0603 (10)	
C4	0.0899 (3)	0.9387 (7)	0.8384 (4)	0.0973 (16)	
H4A	0.1076	0.8772	0.9037	0.171 (14)*	
H4B	0.0388	0.8721	0.8074	0.171 (14)*	
H4C	0.0726	1.0683	0.8529	0.171 (14)*	
B3	0.1682 (3)	0.7752 (7)	0.6574 (3)	0.0651 (12)	
H3	0.1130	0.6699	0.6457	0.078*	
B4	0.2219 (3)	0.8917 (6)	0.5497 (3)	0.0600 (11)	
H4	0.2023	0.8613	0.4667	0.072*	
B5	0.2525 (3)	1.1224 (7)	0.5909 (4)	0.0641 (12)	
H5	0.2533	1.2426	0.5346	0.077*	
B6	0.2166 (3)	1.1519 (6)	0.7254 (4)	0.0655 (12)	
H6	0.1929	1.2898	0.7573	0.079*	
B7	0.2479 (3)	0.7542 (7)	0.7621 (4)	0.0641 (11)	
H7	0.2458	0.6335	0.8182	0.077*	
B8	0.2840 (3)	0.7257 (6)	0.6277 (4)	0.0618 (11)	
H8	0.3061	0.5870	0.5957	0.074*	
B9	0.3368 (3)	0.9424 (6)	0.5869 (3)	0.0569 (10)	
B10	0.3323 (3)	1.1052 (7)	0.6967 (4)	0.0641 (12)	
H10	0.3859	1.2134	0.7098	0.077*	
B11	0.2770 (3)	0.9867 (7)	0.8032 (4)	0.0634 (11)	
H11	0.2940	1.0175	0.8869	0.076*	
B12	0.3516 (3)	0.8587 (7)	0.7199 (4)	0.0654 (12)	
H12	0.4185	0.8061	0.7485	0.078*	
C5	0.4156 (3)	0.9438 (6)	0.5044 (4)	0.0741 (11)	
C6	0.4772 (3)	0.9437 (7)	0.4438 (5)	0.1044 (17)	
H1	0.5260	0.9435	0.3958	0.15 (2)*	

### Atomic displacement parameters (Å^2^)

**Table:**

	*U*^11^	*U*^22^	*U*^33^	*U*^12^	*U*^13^	*U*^23^
C1	0.061 (2)	0.078 (3)	0.058 (2)	0.009 (2)	−0.0032 (19)	0.000 (2)
C3	0.082 (3)	0.156 (5)	0.102 (4)	0.045 (3)	−0.009 (3)	0.001 (4)
C2	0.063 (2)	0.066 (2)	0.052 (2)	−0.0107 (18)	0.0106 (19)	−0.007 (2)
C4	0.090 (3)	0.120 (4)	0.082 (3)	−0.026 (3)	0.033 (3)	−0.013 (3)
B3	0.072 (3)	0.062 (3)	0.061 (3)	−0.020 (2)	0.001 (2)	−0.012 (2)
B4	0.067 (2)	0.070 (3)	0.044 (2)	−0.003 (2)	−0.005 (2)	−0.007 (2)
B5	0.086 (3)	0.056 (2)	0.051 (2)	0.002 (2)	0.005 (2)	0.004 (2)
B6	0.081 (3)	0.053 (2)	0.062 (3)	−0.006 (2)	0.015 (2)	−0.010 (2)
B7	0.091 (3)	0.053 (2)	0.048 (2)	−0.006 (2)	0.002 (2)	0.007 (2)
B8	0.082 (3)	0.047 (2)	0.057 (2)	0.005 (2)	0.005 (2)	−0.001 (2)
B9	0.059 (2)	0.062 (2)	0.050 (2)	−0.003 (2)	0.003 (2)	−0.001 (2)
B10	0.065 (3)	0.067 (3)	0.061 (3)	−0.023 (2)	0.002 (2)	−0.007 (3)
B11	0.075 (3)	0.070 (3)	0.045 (2)	−0.014 (2)	−0.002 (2)	−0.006 (2)
B12	0.061 (2)	0.080 (3)	0.056 (3)	0.005 (2)	−0.009 (2)	0.001 (2)
C5	0.072 (2)	0.081 (3)	0.070 (3)	−0.002 (2)	0.015 (2)	−0.002 (2)
C6	0.094 (3)	0.119 (4)	0.101 (3)	−0.004 (3)	0.042 (3)	−0.003 (3)

### Geometric parameters (Å, °)

**Table:**

C1—C3	1.510 (5)	B5—B9	1.759 (6)
C1—C2	1.680 (5)	B5—B10	1.766 (6)
C1—B4	1.684 (6)	B5—B6	1.778 (6)
C1—B5	1.693 (6)	B5—H5	1.1000
C1—B3	1.714 (6)	B6—B11	1.750 (7)
C1—B6	1.728 (6)	B6—B10	1.751 (6)
C3—H3A	0.9600	B6—H6	1.1000
C3—H3B	0.9600	B7—B11	1.760 (6)
C3—H3C	0.9600	B7—B12	1.757 (6)
C2—C4	1.529 (5)	B7—B8	1.776 (6)
C2—B11	1.670 (6)	B7—H7	1.1000
C2—B7	1.697 (6)	B8—B9	1.777 (6)
C2—B6	1.709 (6)	B8—B12	1.781 (6)
C2—B3	1.717 (5)	B8—H8	1.1000
C4—H4A	0.9600	B9—C5	1.544 (5)
C4—H4B	0.9600	B9—B12	1.780 (6)
C4—H4C	0.9600	B9—B10	1.789 (6)
B3—B7	1.758 (6)	B10—B11	1.765 (7)
B3—B8	1.758 (6)	B10—B12	1.774 (7)
B3—B4	1.760 (6)	B10—H10	1.1000
B3—H3	1.1000	B11—B12	1.752 (6)
B4—B5	1.755 (6)	B11—H11	1.1000
B4—B8	1.767 (6)	B12—H12	1.1000
B4—B9	1.769 (6)	C5—C6	1.175 (6)
B4—H4	1.1000	C6—H1	0.9300
			
C3—C1—C2	118.2 (3)	C2—B6—H6	124.2
C3—C1—B4	121.2 (4)	C1—B6—H6	124.3
C2—C1—B4	110.5 (3)	B11—B6—H6	122.4
C3—C1—B5	122.1 (4)	B10—B6—H6	122.6
C2—C1—B5	110.0 (3)	B5—B6—H6	122.6
B4—C1—B5	62.6 (2)	C2—B7—B11	57.7 (2)
C3—C1—B3	116.9 (4)	C2—B7—B3	59.6 (2)
C2—C1—B3	60.8 (2)	B11—B7—B3	107.4 (3)
B4—C1—B3	62.4 (3)	C2—B7—B12	104.4 (3)
B5—C1—B3	113.5 (3)	B11—B7—B12	59.7 (3)
C3—C1—B6	117.7 (4)	B3—B7—B12	107.8 (3)
C2—C1—B6	60.2 (2)	C2—B7—B8	105.6 (3)
B4—C1—B6	114.0 (3)	B11—B7—B8	108.1 (3)
B5—C1—B6	62.6 (3)	B3—B7—B8	59.7 (3)
B3—C1—B6	112.5 (3)	B12—B7—B8	60.5 (3)
C1—C3—H3A	109.5	C2—B7—H7	124.5
C1—C3—H3B	109.5	B11—B7—H7	122.1
H3A—C3—H3B	109.5	B3—B7—H7	121.6
C1—C3—H3C	109.5	B12—B7—H7	122.4
H3A—C3—H3C	109.5	B8—B7—H7	121.9
H3B—C3—H3C	109.5	B3—B8—B4	59.9 (3)
C4—C2—B11	120.3 (3)	B3—B8—B9	107.8 (3)
C4—C2—C1	119.3 (3)	B4—B8—B9	59.9 (2)
B11—C2—C1	110.7 (3)	B3—B8—B7	59.6 (2)
C4—C2—B7	120.6 (4)	B4—B8—B7	107.5 (3)
B11—C2—B7	63.0 (2)	B9—B8—B7	107.7 (3)
C1—C2—B7	110.3 (3)	B3—B8—B12	106.7 (3)
C4—C2—B6	116.9 (3)	B4—B8—B12	107.2 (3)
B11—C2—B6	62.4 (3)	B9—B8—B12	60.1 (2)
C1—C2—B6	61.3 (2)	B7—B8—B12	59.2 (3)
B7—C2—B6	114.3 (3)	B3—B8—H8	122.2
C4—C2—B3	118.0 (3)	B4—B8—H8	122.0
B11—C2—B3	113.7 (3)	B9—B8—H8	121.6
C1—C2—B3	60.6 (2)	B7—B8—H8	122.1
B7—C2—B3	62.0 (2)	B12—B8—H8	122.5
B6—C2—B3	113.3 (3)	C5—B9—B5	122.1 (3)
C2—C4—H4A	109.5	C5—B9—B4	121.7 (3)
C2—C4—H4B	109.5	B5—B9—B4	59.7 (2)
H4A—C4—H4B	109.5	C5—B9—B8	121.3 (3)
C2—C4—H4C	109.5	B5—B9—B8	107.7 (3)
H4A—C4—H4C	109.5	B4—B9—B8	59.8 (3)
H4B—C4—H4C	109.5	C5—B9—B12	122.6 (3)
C1—B3—C2	58.6 (2)	B5—B9—B12	107.1 (3)
C1—B3—B7	105.9 (3)	B4—B9—B12	107.1 (3)
C2—B3—B7	58.4 (2)	B8—B9—B12	60.1 (2)
C1—B3—B8	105.3 (3)	C5—B9—B10	122.6 (3)
C2—B3—B8	105.5 (3)	B5—B9—B10	59.7 (3)
B7—B3—B8	60.7 (3)	B4—B9—B10	107.3 (3)
C1—B3—B4	58.0 (2)	B8—B9—B10	107.9 (3)
C2—B3—B4	105.2 (3)	B12—B9—B10	59.6 (3)
B7—B3—B4	108.6 (3)	B6—B10—B5	60.7 (3)
B8—B3—B4	60.3 (2)	B6—B10—B11	59.7 (3)
C1—B3—H3	123.9	B5—B10—B11	107.5 (3)
C2—B3—H3	124.0	B6—B10—B12	107.5 (3)
B7—B3—H3	121.6	B5—B10—B12	107.0 (3)
B8—B3—H3	122.5	B11—B10—B12	59.3 (3)
B4—B3—H3	122.2	B6—B10—B9	108.2 (3)
C1—B4—B5	59.0 (2)	B5—B10—B9	59.3 (3)
C1—B4—B3	59.6 (3)	B11—B10—B9	107.4 (3)
B5—B4—B3	108.3 (3)	B12—B10—B9	60.0 (2)
C1—B4—B8	106.2 (3)	B6—B10—H10	121.4
B5—B4—B8	108.3 (3)	B5—B10—H10	122.0
B3—B4—B8	59.8 (3)	B11—B10—H10	122.3
C1—B4—B9	105.7 (3)	B12—B10—H10	122.3
B5—B4—B9	59.9 (3)	B9—B10—H10	121.9
B3—B4—B9	108.1 (3)	C2—B11—B6	59.9 (2)
B8—B4—B9	60.3 (2)	C2—B11—B7	59.2 (3)
C1—B4—H4	123.6	B6—B11—B7	109.2 (3)
B5—B4—H4	121.5	C2—B11—B12	105.8 (3)
B3—B4—H4	121.4	B6—B11—B12	108.5 (3)
B8—B4—H4	121.9	B7—B11—B12	60.0 (3)
B9—B4—H4	122.2	C2—B11—B10	106.2 (3)
C1—B5—B4	58.4 (2)	B6—B11—B10	59.8 (3)
C1—B5—B9	105.8 (3)	B7—B11—B10	108.9 (3)
B4—B5—B9	60.5 (2)	B12—B11—B10	60.6 (3)
C1—B5—B10	105.7 (3)	C2—B11—H11	123.6
B4—B5—B10	109.0 (3)	B6—B11—H11	121.0
B9—B5—B10	61.0 (3)	B7—B11—H11	121.0
C1—B5—B6	59.7 (3)	B12—B11—H11	122.0
B4—B5—B6	108.2 (3)	B10—B11—H11	121.8
B9—B5—B6	108.4 (3)	B11—B12—B7	60.2 (3)
B10—B5—B6	59.2 (3)	B11—B12—B10	60.1 (3)
C1—B5—H5	124.1	B7—B12—B10	108.6 (3)
B4—B5—H5	121.3	B11—B12—B9	108.3 (3)
B9—B5—H5	121.6	B7—B12—B9	108.4 (3)
B10—B5—H5	121.9	B10—B12—B9	60.4 (3)
B6—B5—H5	121.5	B11—B12—B8	108.2 (3)
C2—B6—C1	58.5 (2)	B7—B12—B8	60.3 (2)
C2—B6—B11	57.7 (2)	B10—B12—B8	108.4 (3)
C1—B6—B11	104.8 (3)	B9—B12—B8	59.9 (2)
C2—B6—B10	105.1 (3)	B11—B12—H12	121.6
C1—B6—B10	104.8 (3)	B7—B12—H12	121.3
B11—B6—B10	60.5 (3)	B10—B12—H12	121.3
C2—B6—B5	104.8 (3)	B9—B12—H12	121.5
C1—B6—B5	57.7 (3)	B8—B12—H12	121.6
B11—B6—B5	107.7 (3)	C6—C5—B9	178.2 (5)
B10—B6—B5	60.0 (3)	C5—C6—H1	180.0

## References

[bb1] Brandenburg, K. (2010). *DIAMOND* Crystal Impact GbR, Bonn, Germany.

[bb2] Bregadze, V. I. (1992). *Chem. Rev.* **92**, 209–223.

[bb3] Finze, M. (2008). *Inorg. Chem.* **47**, 11857–11867. 10.1021/ic801610b 18998671

[bb4] Himmelspach, A. & Finze, M. (2010*a*). *Eur. J. Inorg. Chem.* pp. 2012–2024.

[bb5] Himmelspach, A. & Finze, M. (2010*b*). *J. Organomet. Chem.* **695**, 1337–1345.

[bb6] Kalinin, V. N. & Ol’shevskaya, V. A. (2008). *Russ. Chem. Bull.* **57**, 815–836.

[bb7] Oxford Diffraction (2009). *CrysAlis PRO* Oxford Diffraction Ltd, Yarnton, England.

[bb8] Sheldrick, G. M. (2008). *Acta Cryst.* A**64**, 112–122. 10.1107/S0108767307043930 18156677

[bb9] Shmueli, U., Schiltz, M. & Flack, H. D. (2008). *Acta Cryst.* A**64**, 476–483. 10.1107/S0108767308013421 18560164

[bb10] Zakharkin, L. I., Kovredov, A. I. & Ol’shevskaya, V. A. (1981). *Russ. J. Gen. Chem.* **51**, 2422

